# First steps towards a fast-neutron therapy planning program

**DOI:** 10.1186/1748-717X-6-163

**Published:** 2011-11-25

**Authors:** Sylvia Garny, Werner Rühm, Maria Zankl, Franz M Wagner, Herwig G Paretzke

**Affiliations:** 1 Helmholtz Zentrum München, Institut für Strahlenschutz, Ingolstädter Landstraße 1, 85764 Neuherberg, Germany; 2 Klinik und Poliklinik für Strahlentherapie und Radioonkologie, Klinikum der LMU München, Campus Großhadern, Marchioninistraße 15, 81377 München, Germany; 3 Technische Universität München, Forschungsneutronenquelle Heinz Maier-Leibnitz (FRM II), Garching, Germany

## Abstract

**Background:**

The Monte Carlo code GEANT4 was used to implement first steps towards a treatment planning program for fast-neutron therapy at the FRM II research reactor in Garching, Germany. Depth dose curves were calculated inside a water phantom using measured primary neutron and simulated primary photon spectra and compared with depth dose curves measured earlier. The calculations were performed with GEANT4 in two different ways, simulating a simple box geometry and splitting this box into millions of small voxels (this was done to validate the voxelisation procedure that was also used to voxelise the human body).

**Results:**

In both cases, the dose distributions were very similar to those measured in the water phantom, up to a depth of 30 cm. In order to model the situation of patients treated at the FRM II MEDAPP therapy beamline for salivary gland tumors, a human voxel phantom was implemented in GEANT4 and irradiated with the implemented MEDAPP neutron and photon spectra. The 3D dose distribution calculated inside the head of the phantom was similar to the depth dose curves in the water phantom, with some differences that are explained by differences in elementary composition. The lateral dose distribution was studied at various depths. The calculated cumulative dose volume histograms for the voxel phantom show the exposure of organs at risk surrounding the tumor.

**Conclusions:**

In order to minimize the dose to healthy tissue, a conformal treatment is necessary. This can only be accomplished with the help of an advanced treatment planning system like the one developed here. Although all calculations were done for absorbed dose only, any biological dose weighting can be implemented easily, to take into account the increased radiobiological effectiveness of neutrons compared to photons.

## Background

At the former research reactor FRM at Garching, Germany, neutrons have been used for radiation therapy since 1985, and 715 patients with different types of tumors were treated until its shut-down in July 2000. Since June 2007, neutron irradiation treatments are performed at the new Medical Application facility (MEDAPP) at the Research Neutron Source Heinz Maier-Leibnitz (FRM II) [1,2]. This new reactor was designed to provide a very high neutron fluence rate, primarily at thermal energies. In the moderator tank of the reactor, the low-energy neutrons are converted by two uranium plates to high-energy neutrons for medical (and other) applications at beamline SR 10. Filters are used to match the beam characteristics (i.e., neutron spectrum and the neutron-to-photon ratio) to those of the first facility at the former FRM. For this reason, medical knowledge acquired during the operation of the first reactor can be used for the treatment at the FRM II. As main improvements to the earlier facility, the new facility includes a 3-fold increase in total neutron fluence rate and up to a 6-fold increase in field size [3]. This leads to a decrease in treatment time and to an improvement of treatment conditions and quality, which are now comparable to those at other neutron therapy facilities like for example at Seattle, Fermilab or iThemba [4-6].

The next step of improvement would be to introduce a computer-based treatment planning system rather than to use waterphantom depth-dose curves for dose estimation which are used at the moment in a certificated (CE) procedure. The physical basics for such a planning system have already been thoroughly explored both by using the Monte Carlo Code GEANT4 for simulations of neutron and photon transport [7,8], and by using a Bonner sphere spectrometer [9] to measure the neutron energy spectrum at the patient position.

It is noted that in the past, different Monte Carlo algorithms have been applied for dose assessment in radiation therapy. For example, several different programs (including MCNP, MCNPX, EGS4, BEAMnrc VMC, PHITS, GEANT4) were used to simulate photons and electrons [10-16], protons [17-21], neutrons [22,23] and dose from secondary neutrons during proton and ^12^C irradiation [24-27]. GEANT4 also offers the opportunity to calculate the 3D dose distribution of various particles including neutrons, photons and all secondary particles [28]. In addition it allows to include any weighting function for calculation of biologically-weighted doses. This was shown e.g. by [29] to be important for future dose assessment of high LET-irradiation.

This work presents the results of a detailed simulation of water phantom depth-dose curves with GEANT4, based on the measured neutron energy spectrum at the patient treatment position, and its comparison with measurements [30]. First steps towards a patient dose calculation were performed inside a voxel phantom [31] recommended by the International Commission on Radiation Protection (ICRP) for use in radiation protection (ICRP 103/Match 2007). It is shown that the implemented program allows calculation of 3D-distributions of neutron and photon absorbed dose.

## Material and methods

### The Monte Carlo code GEANT4

GEANT4 version 4.8.2 ran on a SuSe-linux system. The neutron-data library G4NDL 3.10 was installed which is based on the ENDF/B-VI cross section evaluation [32]. The required GEANT4-physics list was built including low energy processes. In particular, the S(*α*, *β*)-matrix was implemented to simulate thermal elastic scattering. A detailed description and testing of this physics list is given in [7,33]. The importance of the S(*α*, *β*)-reaction was also discussed by Enger et al [22].

The deposited energy was calculated with a scorer (G4DoseDeposit), which was modified to determine also the statistical error of the dose. The G4SDParticleFilter was applied to score the absorbed dose deposited by photons, electrons, protons and neutrons separately. In order to calculate the neutron fluence rate, an energy-binned G4CellFlux scorer was used, which was also modified to calculate the statistical error. The binning was done with G4SDParticleWithEnergyFilter. Details on the usage of GEANT4 can be found in [34].

### Geometry of the water phantom

**Figure 1:**
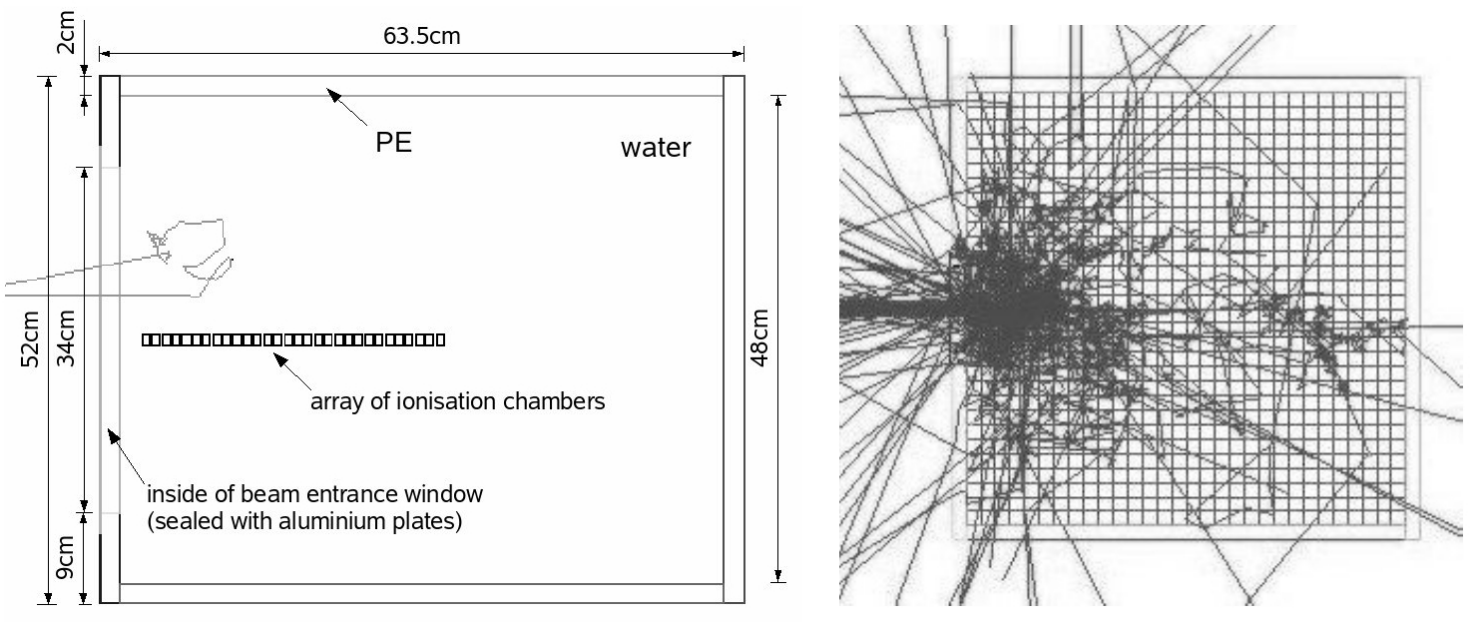
**Geometry of the Water Phantom**. The two geometry types used for the simulation of the water phantom: **left**: solid box phantom with the measurement chambers inside (which are also out of water; 30 of the 40 chambers used in the calculations are shown) with one incoming neutron track; **right**: x-y-plane of the voxelised water phantom with tracks from 100 primary neutrons of the FRM II beam. For visualization purposes, a 2 cm voxel size was used here, instead of the 2 mm used in the actual GEANT4 simulations.

In earlier measurements [1,30] the neutron and photon depth-dose curves in a water phantom were determined by a pair of thimble chambers with different neutron sensitivity that were irradiated at the patient position (EXTRADIN-chamber one out of tissue-equivalent (TE) material A150 with TE gas; sensitivity to neutrons/gamma = 48.5/51.5%; chamber two out of Mg/Ar, neutron sensitivity 2%, chamber volume: 0.5 cm^3^). The phantom consisted of a box of Perspex filled with about 1701 of water with an entrance window for the horizontal beam and a device to position the ionization chambers. This water phantom was simulated here in two different ways (see Figure 1): as a solid box with small measurement chambers inside and in voxelised form consisting of over 10 millions of box-shaped volume elements ("voxels"). Both phantoms consisted of water and were put into a cubic container made of Perspex with dimensions 63.5 × 63.5 × 52 cm^3 ^and 2.0 cm wall thickness (like in the real experiment, material definition see Table1). For the voxelised phantom, the dimensions were slightly enlarged to 64 × 64 × 52 cm^3 ^to simplify the voxelisation algorithm, and voxel sizes of 0.2 × 0.2 × 0.5 cm^3 ^were used in accordance with those used typically in CT-imaging for radiation therapy. The Perspex container also included the entrance window of 12 × 34 cm^2 ^size sealed with two aluminum plates of 1.5 mm thickness.

**Table 1:** Specification of Perspex in GEANT4

atomic composition *	physical quantities
ratio of elements:	temperature = 300 K
C: 5	state of aggregation = solid (kStateSolid)
O: 2	density = 1.19 g/cm^3^
H: 8	for energy loss purposes:
	ChemicalFormula = "(C_2H_4)_N_Polyethylene"

hydrogen in water (TS_H_of_Water) was used for the thermal scattering of neutrons; *natural composition of isotopes

The volume inside the solid water box, where the measurements were performed, was simulated as an array of tube-shaped chambers with a radius of 0.38 cm, a height of 1.21 cm and a volume of 0.54 cm^3 ^that also consisted of water, in order to simulate an undisturbed measurement. Note that after the measurement, the doses measured inside the water phantom by means of the ionization chambers were corrected for the influence of the chamber on the measurement to provide the doses in an undisturbed phantom [3,30]. In the simulation, the chambers were parameterized in such a way that the first chamber was placed at 0.5 cm depth in water, followed every centimeter by another detector chamber. This array of tube-shaped chambers corresponds well with the ionization chamber used for the real measurements (volume 0.5 cm^3 ^[3]). In total there were 40 measurement volumes, which corresponds to a maximum depth of 39.5 cm. In the case of the voxelised phantom, the parameterized chambers were removed and the depth-dose scoring was done directly inside the voxels.

In the voxelised geometry, the total absorbed dose and the absorbed dose from primary and secondary photons and electrons were scored separately, simulating the real measurement, where both the neutron and photon doses were determined. In the case of the solid box geometry it was possible to collect more information, because less scoring volumes were present and therefore less data storage space was needed for each quantity. The total, neutron, proton, electron/positron and photon dose were all scored separately as well as the local moderated neutron fiuence rates in 58 energy bins. In spite of this, the calculation time for the solid box is about 3.5 times faster (4h compared to 14h for 10^6 ^primary neutrons on a 3.2 GHz processor pc).

**Figure 2:**
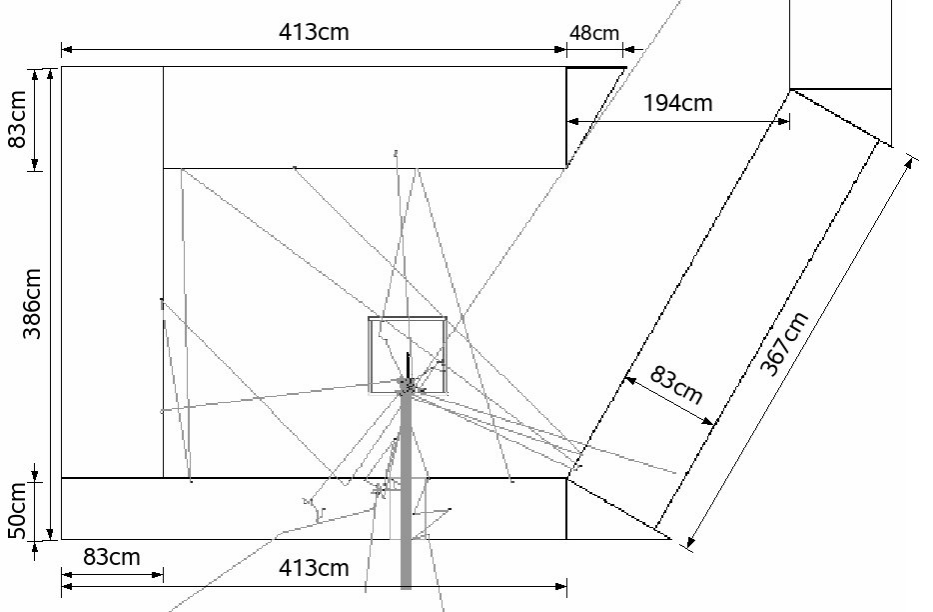
**Geometry of the MEDAPP Room at the FRM II**. Wall material: G4-concrete; max. beam window: 20 × 30 cm^2^; distance to floor/ceiling: 145 cm; thin lines: 10 neutron tracks; thick gray line: neutron therapy beam.

For the solid box geometry, the influence of the surrounding walls, floor and roof of the therapy room was also studied. For this, the patient treatment room was implemented in a schematic way, using G4-concrete from the NIST-material database as wall material [35]. The walls were simulated to be 83 cm thick (50 cm in case of the beam exit wall) with a beam exit window of 20 × 30 cm^2^. In Figure 2, the top-view of the MEDAPP therapy room as simulated in GEANT4 is shown together with 10 neutron tracks using a neutron spectrum that had been measured by means of a Bonner sphere spectrometer at the patient position [9,33].

In both geometries, the primary neutron and photon beam cross section was a square of 9 × 9 cm^2^, which is the same as the one used for the measurements [1,3].

### Geometry of the human voxel phantom

The human voxel phantom used here is based on CT-scans of a real person. The Hounsfield numbers of the CT-slices were translated to 141 organ-IDs by hand, using anatomical atlases [31]. Each voxel has the size 1.775 × 1.775 × 4.84 mm^3 ^(x, y, z-direction), with the long side of the voxel aligned along the z-axis of the voxel phantom (increasing from toe to head). In total, there are 299 columns, 137 rows and 337 slices (x, y, z-direction) of voxels in the phantom, with vacuum-filled voxels surrounding the human body. The phantom represents an idealized female of 163 cm height and 60 kg body mass, based on the voxel phantom segmented from the CT-scan of a woman (Laura: 167 cm height, 59 kg). Compared to the original phantom Laura, the voxel size was changed, and the masses of individual organs were adjusted to reference values, to fulfill the requirements of ICRP 89 [36], using anatomical books for guidance. In this way, the Reference Female REGINA that was used in the present task was constructed.

Because the application of the FRM II neutron beam is most promising for the head and neck region, only the upper quarter of the voxel phantom (including 87 slices) was used for the calculations. The orientation of the voxel phantom in GEANT4 is the following: the column numbers (x-direction) increase from the right to the left side of the phantom, the row numbers (y-direction) increase from the nose to the back side of the head, and the slice numbers (z-direction) increase from the breast to the head. The voxel phantom was implemented in GEANT4 using the fast phantom parameterization (imported from version 4.9.0 into the used version 4.8.2), which includes all voxels (also the surrounding vacuum voxels) and identifies a specific voxel by its position in the grid. This algorithm is very fast but requires a lot of memory because the scored information is saved for all voxels that are placed.

The material of the voxel at (x, y, z)-position is set according to the number given in the phantom's datafile: the 141 organ IDs are projected onto 30 different materials (with arbitrary numbers) which are then used in the calculations (see [31] for details on the atomic composition and organ-to-material conversion).

**Figure 3:**
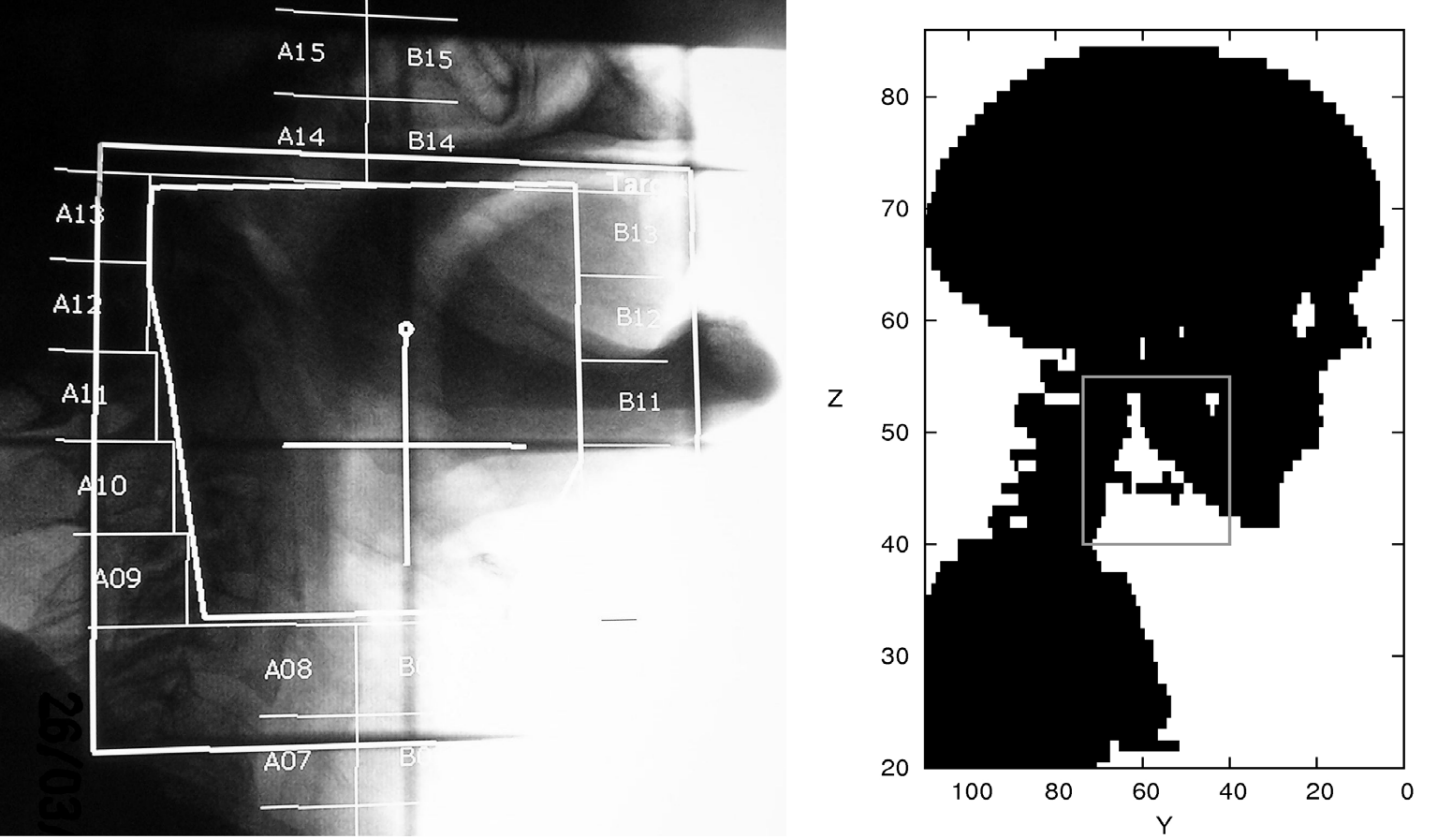
**Beam Position and Size**. Comparison of beam position and size for a real patient treatment (left panel; Loeper, MRI/TUM, priv. com.) and the simulated field in the human voxel phantom (gray lines in right panel).

The chosen test case was a salivary gland treatment of the right submandibular gland (lower jaw salivary gland). Because the real field size applied to the patient was almost a rectangle (see left side of Figure 3, a rectangular beam with 6 cm × 7 cm cross section was used in the simulations hitting the patient from the right side with a beam direction along the positive x-axis. In contrast to the real situation, the beam profile was also simulated to be rectangular (see next section ). The position of the beam was simulated in resemblance to that of the real patient case using the skeletal structure as a guideline. It should be emphasized, however, that some approximations had to be made because the head-to-body angle of the voxel phantom was somewhat different to that of the real case (see Figure 3). In this way, the calculation algorithm could be tested and a first assessment of the absorbed dose distribution was possible. It should be noted that the field shape can easily be changed in the simulation to adapt it to the shape of the planning target volume (PTV) if necessary.

### Primary neutron and photon spectrum

**Figure 4:**
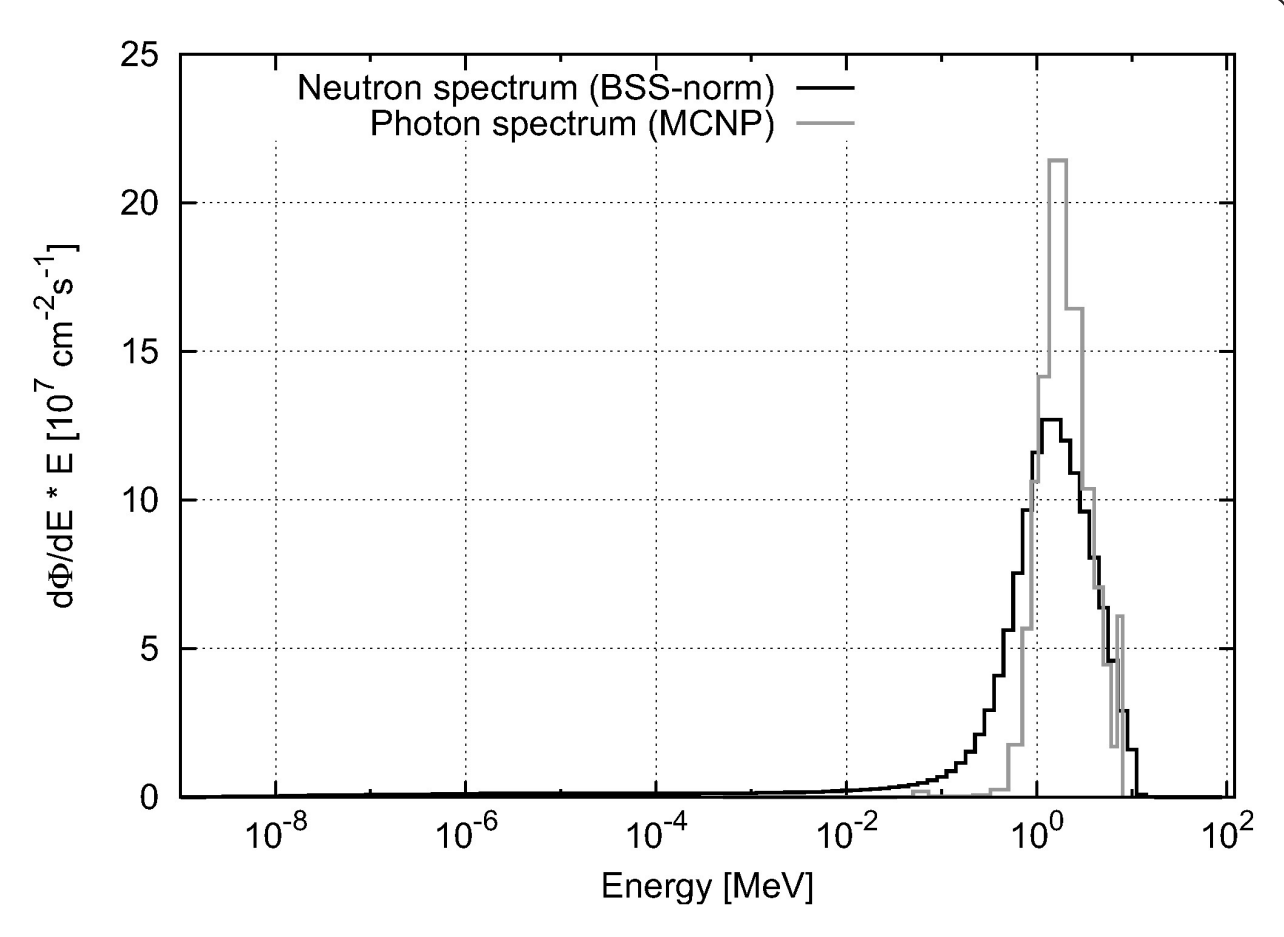
**Neutron and Photon Spectrum**. Neutron and photon spectrum with total particle fluence rate of 3.2 · 10^8 ^cm^-2^s^-1 ^and 2.9 · 10^8 ^cm^-2^s^-1^, respectively; neutron spectrum measured with BSS [9]; photon spectrum calculated with MCNP by [38].

The absorbed dose rate (in Gy/s) was determined in GEANT4 by multiplication of the calculated absorbed dose deposited inside the chamber or voxel per primary neutron or photon fluence (resulting in a value with the unit Gycm^2^) with a total primary neutron fluence rate of 3.2 · 10^8 ^cm^-2^s^-1 ^and a total primary photon fluence rate of 2.9 · 10^8 ^cm^-2^s^-1^. The primary neutron fluence rate had independently been determined before by gold-foil activation measurements in a water phantom [37] and by Bonner sphere measurements rescaled to the patient treatment position in the MEDAPP therapy room [9], while the primary photon fluence rate was derived from the comparison of calculated and measured depth-dose curves in water (see discussion below). To get the absolute absorbed dose for a specific treatment field inside the voxel phantom, the resulting values were finally multiplied by the actual irradiation time. In Figure 4, the neutron (measured by a BSS spectrometer [9]) and photon (calculated with MCNP [38]) primary spectra are normalized to a total fluence rate of 3.2 · 10^8 ^cm^-2^s^-1 ^for neutrons and 2.9 · 10^8 ^cm^-2^s^-1 ^for photons, respectively.

In order to sample the neutrons and photons in the calculation according to the measured/calculated spectra, these spectra were integrated and normalized. From the resulting probability function, the primary neutrons and photons were sampled using random numbers.

The primary neutrons and photons were simulated to be homogeneously distributed over the whole beam. Furthermore, the beam profile was approximated to be rectangular with no decrease towards the edges. This is a simplification as the real beam is spread because of neutron scattering inside the beam tube and the large lateral dimensions of the source. Therefore, the real beam has a shallow decline towards the edges [37].

## Results and Discussion

### Calculated depth-dose curves in the water phantom

#### Neutrons

**Figure 5:**
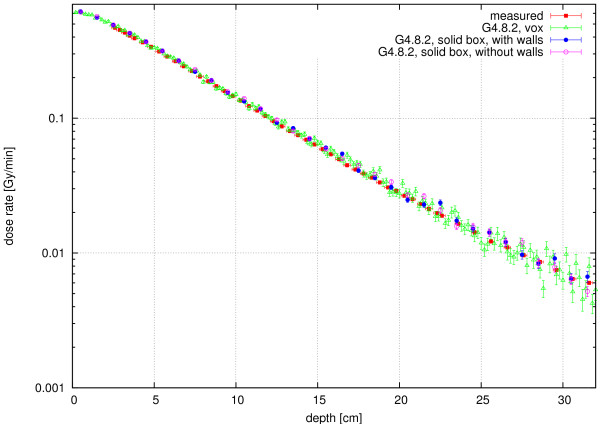
**Neutron Dose Rate**. Neutron dose rate in the water phantom (beam profile of 9 × 9 cm^2 ^): comparison of the voxelised (green triangle, integration over 8 central voxels) and the solid box geometry (blue circle = with walls, pink circle = without walls) calculated with GEANT4 (error bars provide one standard deviation as calculated in GEANT4), together with the measurement of Kampfer [30] (red square).

In Figure 5, the calculated absorbed dose rates in the solid-box and the voxelised geometry are shown together with the measured depth dose curve. The results of the solid box and the voxelised geometry are in excellent agreement. The latter were integrated over 4 voxels in lateral and 2 voxels in vertical direction around the central beam axis, resulting in a dose collection volume of 0.16 cm^3 ^from 8 voxels, to get better statistics. Figure 5 demonstrates that the voxelisation algorithm produces the same results as the solid-box calculation.

Figure 5 also demonstrates the excellent agreement of the calculations with the measurements. For example, the decrease of the dose rate with depth is very similar for both cases. Small differences between the measured and the calculated curves in terms of their absolute values could be - among other reasons - due to the different date of the measurement (the Bonner sphere measurements were performed approximately 1 year after the depth dose measurements, resulting in a small converter plate burn-up of approximately 2% [37]) and, for greater depths, due to calculation statistics. Furthermore, changes in the humidity of the air in the treatment room and the beam tube as well as the exact position of the control rods have an influence on the neutron spectrum and therefore can also change the deposited dose.

Finally, the comparison between the depth dose curves calculated with and without the concrete walls of the treatment room (Figure 5) demonstrate the influence of these walls to be of minor importance. For example, for small and medium depths (up to 10 cm) it is less than 2%. Thus, it is not necessary to consider the treatment room when calculating the dose inside the beam in the voxel phantom.

#### Photons

The primary FRM II treatment beam also includes a photon component, which is caused by prompt gammas during fission, by delayed gammas from fission products in the converter plate, by gammas from activated structures and by Compton scattered gammas from the reactor core. This primary photon spectrum is not yet characterized experimentally. However, an MCNP calculation has already been performed [38], transporting the photons through the beam tube up to the patient treatment position. With this program, it was not possible to simulate exactly the production of photons from the decay of activation and delayed fission products in the converter plates: The simulation resulted in an overall photon fluence rate which was 40% too low, and in a poor reliability of the photon spectrum. Nevertheless, this spectrum was used as a first estimate, to obtain information on the photon depth dose distribution inside the phantom.

For the calculations inside the water phantom, both the solid box and voxelised geometry were used (with and without surrounding walls), and the results compared to the measured data [1]. It should be noted that the measured photon dose rate also includes contributions from secondary photons produced by the neutrons inside the water phantom, as the twin method applied in the measurements does not distinguish between primary and secondary photons.

**Figure 6:**
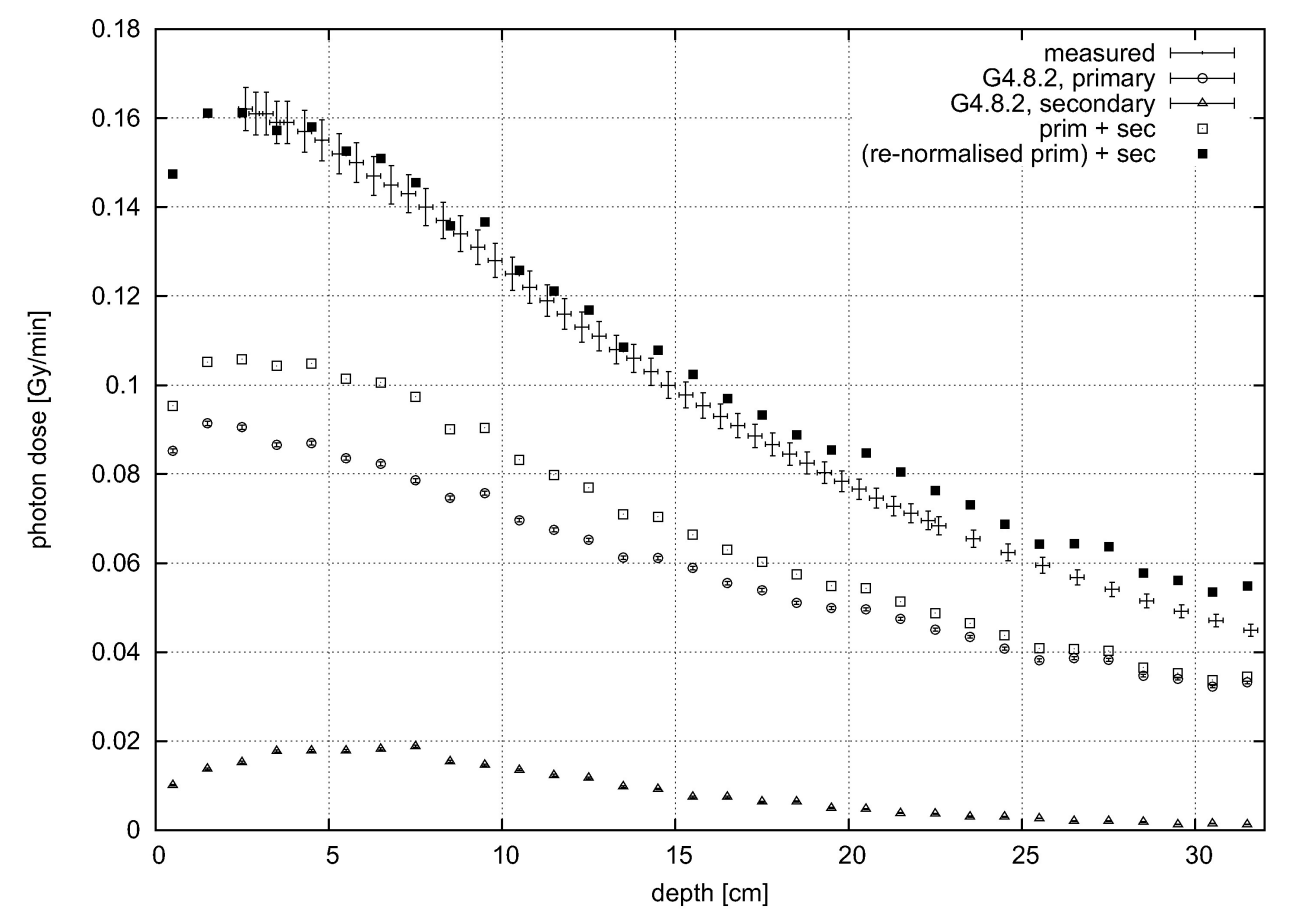
**Primary and Secondary Photon Dose Rate**. Primary and secondary photon dose rate calculated in the solid-box water phantom (error bars provide one standard deviation as calculated in GEANT4), in comparison to measured [30] data (beam profile of 9 × 9 cm^2^); □ = total dose rate from primary (Δ) and secondary (○) photons; ■ = total dose rate from the re-normalized dose rate of primary photons together with that of the secondary photons. Note that while the absolute uncertainties seem to decrease with depth, the relative uncertainties increase with depth.

As for the neutron calculations, the photon calculations in the voxelised and solid-box geometry produce very similar results. Furthermore and similar to the neutron case, inclusion of the surrounding walls does not influence the calculated dose rates. In Figure 6, both the dose rate from primary and secondary photons which were produced by interaction of primary neutrons with the phantom material, are shown. Because the depth-dose curve of the neutrons is consistent with the measured data both in relative and absolute terms (see previous section), the dose rate calculated for secondary photons produced by neutrons is also correct on an absolute scale. On average, the contribution from the secondary photons amounts to about 10-15% of the total measured photon dose rate in the first centimeters. The total primary photon fluence rate used in the simulations (i.e., 1.8 · 10^8 ^cm^-2^s^-1^, calculated by integrating the photon spectrum) is not correct on an absolute scale (see discussion above) but had to be increased until the sum of the calculated depth dose curves of the primary and secondary photons matched the measurements in the first 10 cm, and a best estimate of the primary photon fluence rate of 2.9 · 10^8 ^cm^-2^s^-1 ^was obtained which agrees well with the expected total photon fluence rate (see above). This means that the incident primary total fluence rates of photons and neutrons are rather similar. Though the primary photon spectra is expected to be softer, the shapes of the depth dose curves do not deviate much in the first 10 cm (see Figure 6). Therefore, it is concluded that the primary photon spectrum derived here can be used together with the renormalized total fluence rate for first calculations in the voxel phantom.

### Calculated doses in the voxel phantom REGINA

#### Depth-dose curves and lateral distribution

**Figure 7:**
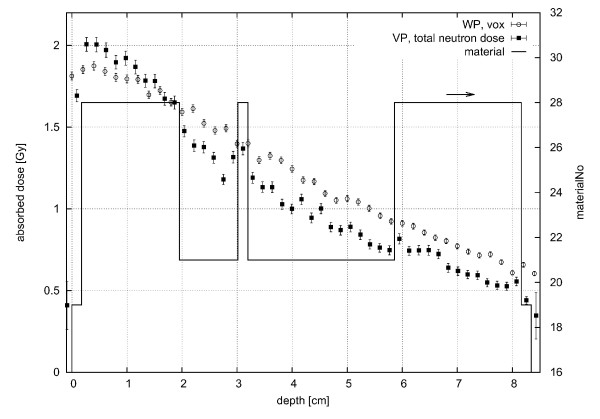
**Absorbed Depth-Dose Curves**. Absorbed depth-dose curves for the FRM II neutron beam in the REGINA phantom, at slice number 45 and row number 48 (beam center; ■), assuming a rectangular beam profile of 6 × 7 cm^2^, and in the voxelised water phantom (○); All curves were calculated for 3 min irradiation with a primary neutron fluence rate of 3.2 · 10^8^n/cm^2^s (no primary photons); for comparison, material numbers in the REGINA phantom along the central beam are included on the right y-axis (19: skin, 21: muscle, 28: adipose tissue).

**Figure 8:**
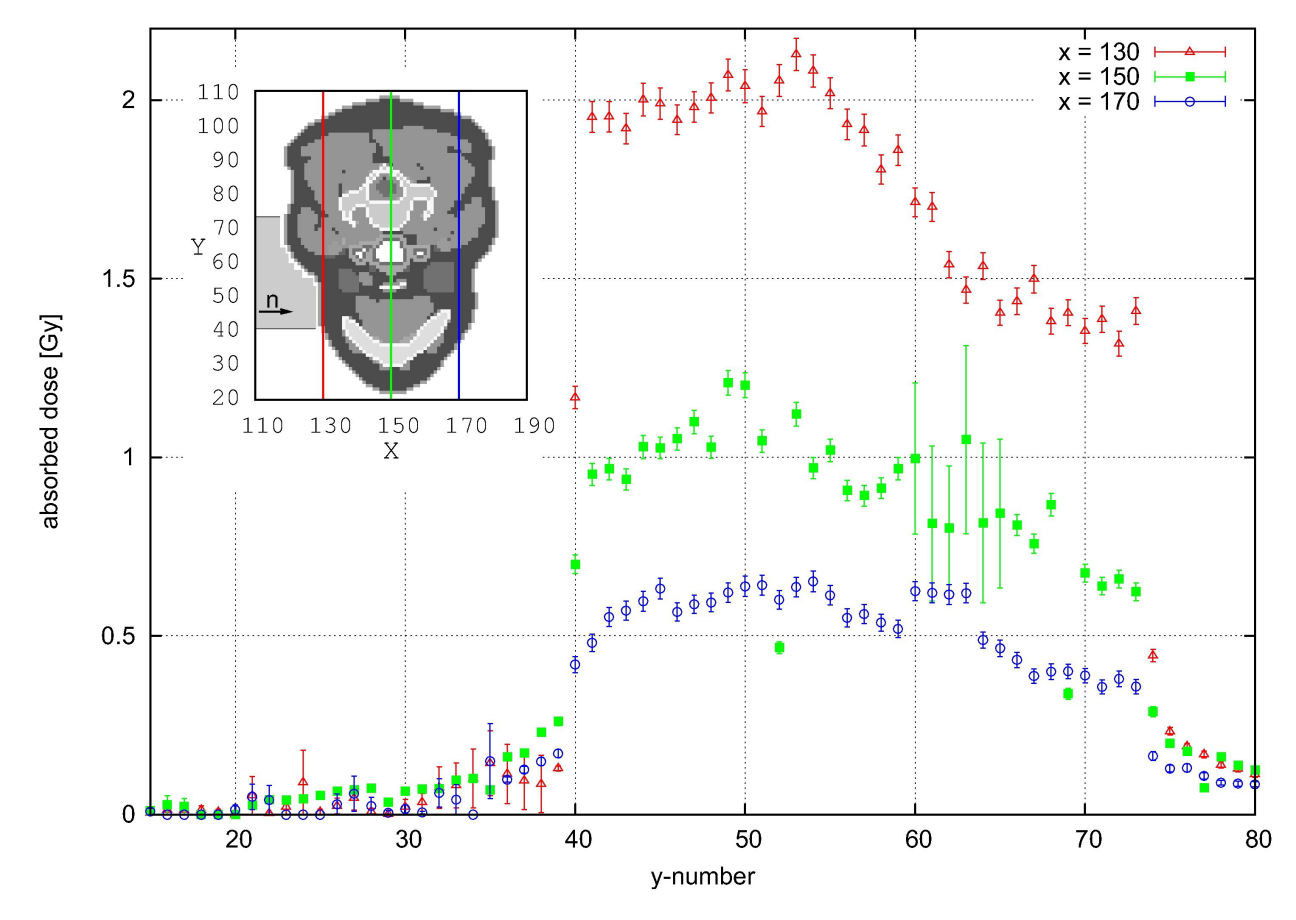
**Absorbed Lateral Dose Distribution**. Absorbed lateral dose distribution of the 6 × 7 cm^2 ^FRM II neutron beam in different depths inside the REGINA voxel phantom after 3 minutes of irradiation with a total primary neutron fiuence rate of 3.2 · 10^8^n/cm^2^s (no primary photons) in slice (z) number 44 assuming a rectangular lateral beam profile.

In Figure 7, the calculated depth-dose curve along the central beam is compared to that obtained in the water phantom. The decrease of dose with depth is similar for both phantoms, but slightly steeper for the voxel phantom, because its atomic composition is different from water (for example, the hydrogen content in adipose tissue (material number 28, see section about the geometry of the voxel phantom ) is 62.5% compared to 66.7% in water). The change of material with depth in the REGINA phantom is also indicated in the figure. When the material changes, e.g., from 19 (skin) to 28 (adipose tissue) or to 21 (muscle), the atomic composition changes and therefore a change in the dose can also be seen. In Figure 8, the lateral absorbed dose distribution is shown for three different columns inside the REGINA voxel phantom: x = 130, 150 and 170, which is equivalent to 3.55 cm distance in between. The neutrons are significantly scattered inside the REGINA voxel phantom and deposit their energy not only inside the beam profile but also in the surrounding tissue. This can be seen at a row (y)-value of 40, where the sharp edges of the simulated rectangular beam can still be seen for small depths (x = 130 is equal to 0.3 cm depth at this y-value) inside the phantom. These sharp edges are washed out with depth, so that in greater depths (e.g. x = 170 or 7.4 cm), the lateral dose is spread over several centimeters (the relative decrease in absorbed dose between y = 39 and y = 41 compared to the dose at y = 41 is 93% for x = 130, 73% for x = 150 and 64% for x = 170). At a y-value of about 74, the neutrons have already passed through a certain thickness of tissue (i.e., x = 130 is equal to 2.0 cm depth at this y-value) and the changes of the lateral spread at the same x-values is less prominent (relative decrease in absorbed dose between y = 73 and y = 75 is 83%/68%/64% for x = 130/150/170, respectively). Note that the beam spread is expected to be even more pronounced for the real beam, because the beam divergence of 1-2° as well as the penumbra of the different filters and collimators were not included in the calculations, but would further increase the lateral spread of the beam (see the discussion in the section about the neutron and photon spectra). Furthermore, the influence of the material is visible in Figure 8 as well. At the same x-value, the dose declines towards higher y-values, which correspond to the back side of the phantom. This is caused by the phantom's uneven surface. Looking at the relevant slice (z = 44, see inset), it can be seen that the neck starts to bulge in the relevant area. Therefore, the radiation is absorbed before reaching the relevant depth whereas at lower y-values (40-55), less material is present. For the green data points (x = 150) an area of high statistical uncertainty can be seen between y-values of 60 to 66. This is the area where the trachea is located, where fewer particles interact with the air inside and deposit dose there. Behind this area, more energy is therefore deposited which can indeed be seen in the data of (x = 170). Another influence of material can for example be seen at y = 35, 52, 69, 77 in depth x = 150, where the deposited dose is much smaller than in the surrounding voxels. The material at this position is hard bone (cortical), with a hydrogen content of only 3.5% (fractionmass) compared to more than 8 for all other soft matter (also bone marrow and spongiosa). Because the neutron dose deposition mainly depends on the hydrogen content of the present material, the dose in these voxels is about half of the dose in other voxels.

#### Dose volume histograms

**Figure 9:**
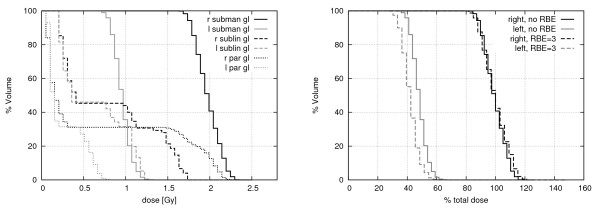
**Dose-Volume-Histogram**. Cumulative (left) dose-volume-histogram (DVH) of the salivary glands after 3 min irradiation with the 6 × 7 cm^2 ^FRM II neutron beam, a total primary neutron fluence rate of 3.2 · 10^8 ^n/cm^2^s and a total primary photon fluence rate of 2.9 · 10^8 ^7/cm^2^s; r = right, 1 = left; subman gl = submandibular gland; subli gl = sublingual gland; par gl = parotid gland. Right histogram: dependence of DVH of right and left submandibular glands on RBE, dose normalized to dose at 50% volume of the right/left submandibular gland respectively.

In patient treatment planning, a tool used for plan quality assessment is the dose-volume-histogram (DVH). In this histogram, the detailed spacial data is condensed into a plot with the fraction of volume of an organ irradiated with a certain dose on the y-axis and the corresponding absorbed dose on the x-axis. In the left panel of Figure 9, the cumulative DVH of the studied salivary gland case is shown for six organs: the healthy salivary glands of the REGINA phantom's left side, the treated right submandibular gland, and the (healthy) right sublingual and parotid gland which are also partly in the beam. It should be emphasized that in a real case, the neutron treatment is given mostly as a boost after a normal photon treatment at a linear accelerator. If the doses from the photon treatment were included, the DVH would change significantly. Furthermore, only the physical absorbed dose was considered here without biological weighting of the neutron dose. If such a biological weighting was included, the DVHs would also change because of the different fraction of dose deposited by photons, depending on the organ's position inside the body.

The right panel of Figure 9 shows the dependence of the DVH of the two submandibular glands on the inclusion of the RBE. For this qualitative analysis, a clinical RBE of three (derived from old clinical data (Wagner, priv com.)) for the neutrons was included after the Monte Carlo calculation, to estimate the influence of a biological weighting for neutrons and their secondary particles. However, at the current state of investigation a proper RBE as a function of neutron energy is not available. The fraction of dose deposited by photons is larger for the left than for the right submandibular gland. Therefore, the ratio of the weighted dose compared to the absorbed dose is larger for the right submandibular gland than for the left one. This reduces the effective dose in the healthy submandibular gland and similarly in other deeper-located organs.

#### Isodoses

**Figure 10:**
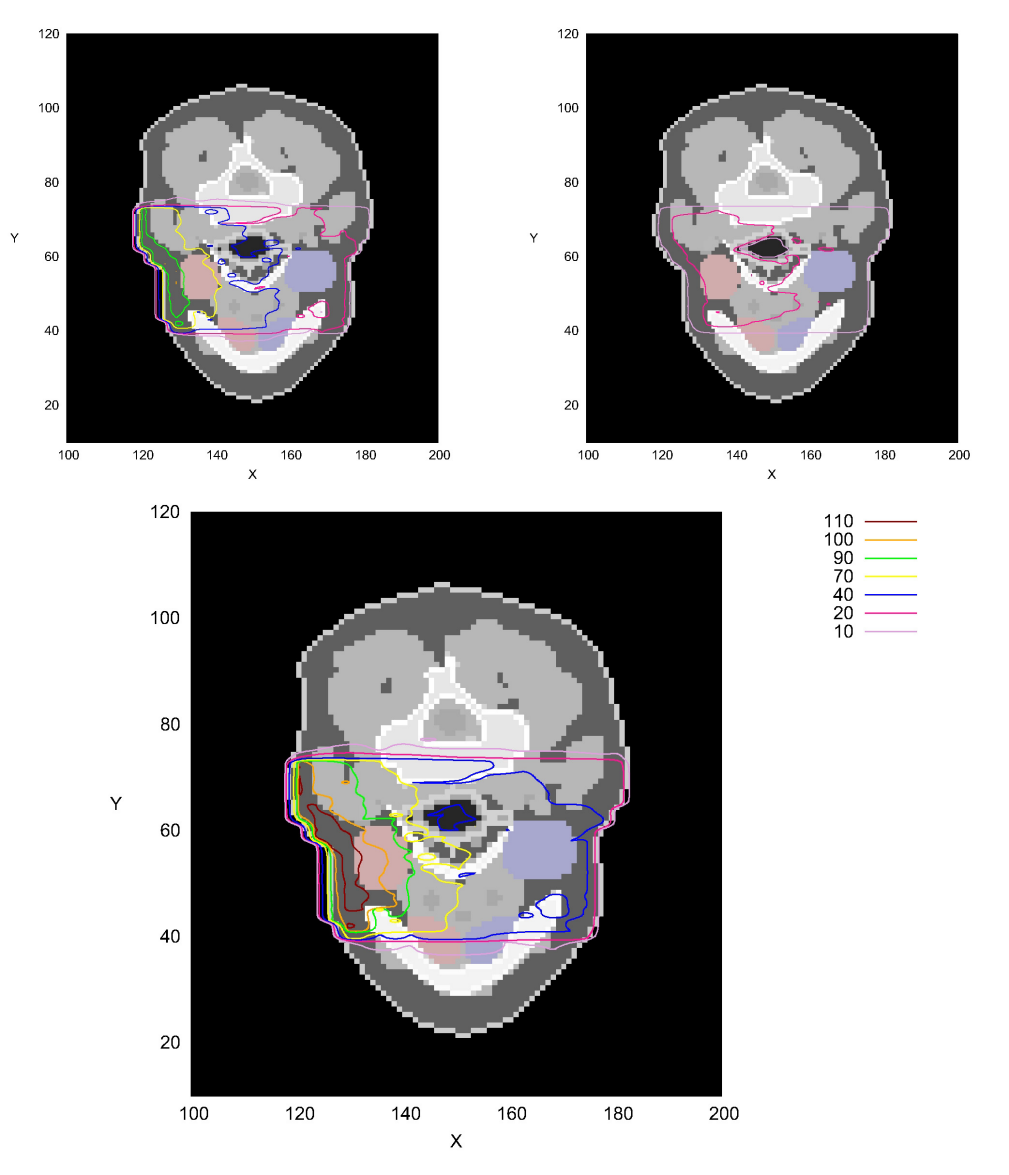
**Absorbed Dose inside REGINA Voxel Phantom**. Absorbed dose inside REGINA voxel phantom after 3 min irradiation with the 6 × 7 cm^2 ^FRM II beam, a total primary neutron fluence rate of 3.2 · 10^8^n/cm^2^s and a total primary photon fluence rate of 2.9 · 10^8^n/cm^2^s; all contour lines relative to the prescribed dose of 2 Gy (in %); slice 45; in red/blue, the right/left salivary glands are highlighted; **top left**: dose from primary neutrons; **top right**: dose from primary photons; **bottom**: total dose; No biological weighting for the neutrons was applied in the figure.

In the top left panel of Figure 10, one slice of the voxel phantom is shown together with the isodose lines of the irradiation with the FRM II neutron spectrum. The isodose lines were achieved by a spline interpolation of the calculated dose-matrix. In hard bone material, lower doses can be observed which may be due to the lower hydrogen content (31.2% element abundance compared to 62.5% in adipose tissue). Inside air cavities such as the trachea, the deposited dose is also somewhat reduced, due to the low density and the lack of hydrogen. This can be seen in the middle part of the slice. In the top right panel of Figure 10, the primary photon absorbed dose with depth is shown, while the lower panel shows the total absorbed dose from primary neutrons and photons (here neutrons are not weighted for their increased biological effectiveness compared to that of photons). It can be seen that the total absorbed dose is about 0.5 Gy higher with the primary photons than without. This is particularly important for the healthy left salivary gland, because the depth-dose curve of the photons is very shallow. This effect leads to an increased absorbed dose to healthy tissues behind the tumor. Introducing the real distribution of neutrons and photons (including the beam spread) would not change this conclusion significantly. It should be noted here that the biological effectiveness of neutrons is very important to assess the dose in a real clinical case.

## Conclusions

The absorbed depth dose distribution of the primary neutrons and photons of the FRM II beam was calculated using GEANT4 in a voxelised water phantom, and a very good agreement with measured data was found. The voxel-algorithm of GEANT4 was validated and influences of the surrounding walls on the dose in the main beam were studied.

The voxel phantom REGINA [31] was used as a first test and a 3D dose distribution of a salivary gland was calculated. The depth dose curve obtained was compared to that in the water phantom and the lateral distribution in different depths was discussed in detail. Furthermore, the dose volume histogram of this test case was calculated and the isodose representations in one slice given as an example. It is emphasized that the calculations described in the present work were done using a parallel neutron beam impinging on the human voxel phantom. Any allowance for beam divergence is expected to modify the calculated isodose lines and DVHs somewhat. Thus, before any more realistic 3D distributions can be given for an individual patient, the beam characteristics and its influence on the 3D dose distribution within a patient should be investigated in detail. In this respect, the given dose distributions (see Figures 7, 8, 9 and 10) should be interpreted still with care. Nevertheless, it is noted that the given dose distributions represent a major step forward in planning of fast-neutron therapy at FRM II, as they are based on quite realistic neutron and photon input spectra, and a realistic human morphology. Note that, to the best of our knowledge, GEANT4 has never been used for this application, and so far only simple water phantoms are being used in the certified dose estimate procedure applied at the MEDAPP facility.

GEANT4 allows to assess a biological dose using radiation weighting factors. Such factors can include particle type, energy and energy loss as well as dose deposition or other quantities like oxygen content or radiation sensitivity of the tissue involved. For the case of chromosomal aberrations Schmid et al. [39] have shown that the irradiation is more effective in the first few centimeters of tissue compared to deeper regions. Similar results were obtained by Magaddino et al. [40] for the tumor control probability and the RBE for permanent colony control. The effect was qualitatively shown in this paper by applying a clinical RBE and could also be seen when artificial RBEs were implemented in the Monte Carlo calculation [33,41]. This helps to lessen the problem of high doses in healthy organs on the patient's side which is opposite to the beam. In the future, cell experiments like [42-44] will be used to improve the biological weighting of the dose.

It is concluded that the results presented here represent a significant step further towards a reliable, patient-specific treatment planning system at the fast-neutron therapy facility MEDAPP. However, further work needs to be done including modeling of beam divergence and exploring the influence of patient-specific parameters on organ doses, before more accurate patient-specific organ doses can be calculated. It is also planned in the future to apply the present approach to physical humanoid phantoms.

## Competing interests

The authors declare that they have no competing interests.

## Authors' contributions

SG: Writing code, running the calculations, producing the outcome, writing Manuscript, WR: Discussing outcome at various stages of the project and critical revision of the manuscript, MZ: Constructing and providing voxel phantom, FMW: providing details in photon and neutron FRM II spectrum, helping with FRMII questions and revision of the manuscript, HGP: Development of the concept, discussion of the outcome and critical revision of the manuscript. All authors read and approved the final manuscript.
